# Identification of transforming growth factor-beta-regulated microRNAs and the microRNA-targetomes in primary lung fibroblasts

**DOI:** 10.1371/journal.pone.0183815

**Published:** 2017-09-14

**Authors:** Jennie Ong, Wim Timens, Vijay Rajendran, Arjan Algra, Avrum Spira, Marc E. Lenburg, Joshua D. Campbell, Maarten van den Berge, Dirkje S. Postma, Anke van den Berg, Joost Kluiver, Corry-Anke Brandsma

**Affiliations:** 1 University of Groningen, University Medical Center Groningen, Department of Pathology and Medical Biology, Groningen, The Netherlands; 2 University of Groningen, University Medical Center Groningen, Groningen Research Institute for Asthma and COPD (GRIAC), Groningen, The Netherlands; 3 Boston University, School of Medicine, Department of Medicine, Section of Computational Biomedicine, Boston, Massachusetts, United States of America; 4 University of Groningen, University Medical Center Groningen, Department of Pulmonary Diseases, Groningen, The Netherlands; National Institute of Technology Rourkela, INDIA

## Abstract

**Background:**

Lung fibroblasts are involved in extracellular matrix homeostasis, which is mainly regulated by transforming growth factor-beta (TGF-β), and are therefore crucial in lung tissue repair and remodeling. Abnormal repair and remodeling has been observed in lung diseases like COPD. As miRNA levels can be influenced by TGF-β, we hypothesized that TGF-β influences miRNA expression in lung fibroblasts, thereby affecting their function.

**Materials and methods:**

We investigated TGF-β1-induced miRNA expression changes in 9 control primary parenchymal lung fibroblasts using miRNA arrays. TGF-β1-induced miRNA expression changes were validated and replicated in an independent set of lung fibroblasts composted of 10 controls and 15 COPD patients using qRT-PCR. Ago2-immunoprecipitation followed by mRNA expression profiling was used to identify the miRNA-targetomes of unstimulated and TGF-β1-stimulated primary lung fibroblasts (n = 2). The genes affected by TGF-β1-modulated miRNAs were identified by comparing the miRNA targetomes of unstimulated and TGF-β1-stimulated fibroblasts.

**Results:**

Twenty-nine miRNAs were significantly differentially expressed after TGF-β1 stimulation (FDR<0.05). The TGF-β1-induced miR-455-3p and miR-21-3p expression changes were validated and replicated, with in addition, lower miR-455-3p levels in COPD (p<0.05). We identified 964 and 945 genes in the miRNA-targetomes of unstimulated and TGF-β1-stimulated lung fibroblasts, respectively. The TGF-β and Wnt pathways were significantly enriched among the Ago2-IP enriched and predicted targets of miR-455-3p and miR-21-3p. The miR-455-3p target genes *HN1*, *NGF*, *STRADB*, *DLD* and *ANO3* and the miR-21-3p target genes *HHEX*, *CHORDC1* and *ZBTB49* were consistently more enriched after TGF-β1 stimulation.

**Conclusion:**

Two miRNAs, miR-455-3p and miR-21-3p, were induced by TGF-β1 in lung fibroblasts. The significant Ago2-IP enrichment of targets of these miRNAs related to the TGF-β and/or Wnt pathways (*NGF*, *DLD*, *HHEX*) in TGF-β1-stimulated fibroblasts suggest a role for these miRNAs in lung diseases by affecting lung fibroblast function.

## Introduction

Lung fibroblasts play a key role in extracellular matrix (ECM) homeostasis and maintenance of the normal lung architecture and are therefore crucial players in lung damage and repair. They have been postulated as important players in lung diseases with disturbed ECM homeostasis and aberrant repair such as chronic obstructive pulmonary disease (COPD). Transforming growth factor beta (TGF-β), an important cytokine in tissue repair and remodeling, stimulates ECM production by fibroblasts mainly through signaling via the downstream SMAD proteins [1–3]. In addition to stimulation of ECM production, this multifunctional cytokine regulates ECM homeostasis by influencing the expression and activity of matrix-metalloproteinases and their inhibitors [4].

MicroRNAs (miRNAs) have been shown to play a key role in the regulation of cellular activity. MiRNAs are small non-coding RNAs with an average length of 22 nucleotides. They are estimated to regulate over 60% of the protein-coding genes [5]. MiRNAs regulate gene expression by partially binding to complementary homologous sequences present on their target mRNA transcripts which then leads to either inhibition of protein translation or mRNA degradation.

The TGF-β signaling pathway is involved in the maturation of a subset of miRNAs, indicating that the levels of several miRNAs might be influenced by TGF-β [6]. SMAD proteins are able to mediate the maturation of miRNAs [7] and can indirectly regulate transcription of miRNAs with a SMAD-binding element in their promoter [8, 9]. Conversely, several miRNAs have been reported to influence TGF-β signaling by regulating the expression of TGF-β1, TGF-β receptors 1 and 2, SMAD1-5 and SMAD7 [6, 10, 11].

COPD is one of the lung diseases with, mostly smoking-induced, disturbed repair capacity of the lung, resulting in emphysematous lung tissue destruction and (small) airway wall fibrosis [12]. TGF-β levels are higher in COPD patients than in control subjects, underscoring the importance of the TGF-β signaling pathway in COPD [12, 13].

Several miRNAs have been found to be deregulated in lung tissue, serum or sputum of COPD patients (reviewed by Osei et al. [14]). The expression level of miR-146a was decreased in IL-1β/TNF-α-treated primary lung fibroblasts of COPD patients compared to those of control subjects [15]. Moreover, altered levels of circulating miRNAs have been proposed as potential biomarkers for COPD [16].

To our knowledge, only limited information is available regarding the interplay between miRNAs and the TGF-β-induced repair response in primary lung fibroblasts, nor is there information on the actual target gene repertoire of miRNAs (i.e. the miRNA-targetome) in these cells. Therefore, the aim of the current study was to investigate the TGF-β1-induced miRNA expression changes in primary lung fibroblasts and to identify the transcripts that are likely to be affected by these miRNAs using the lung fibroblast miRNA-targetomes in unstimulated and TGF-β1-stimulated primary lung fibroblasts. Furthermore a comparison was made between control and COPD fibroblasts, being one of the lung diseases in which ECM remodeling plays an essential role.

## Materials and methods

### Subjects

Parenchymal lung fibroblasts from 9 current or ex-smoking control subjects, undergoing lung surgery for tumor resection were subjected to miRNA profiling using a microarray approach. These primary fibroblasts were isolated from parenchymal lung tissue that was located far away from the tumor and lacked abnormalities as checked by histology on haematoxylin and eosin stained slides [17, 18]. The replication study group consisted of parenchymal fibroblasts from 10 control subjects and 15 COPD patients and was used to replicate the array findings and to investigate the miRNA expression in parenchymal fibroblasts of COPD patients. The controls had no history of lung disease, other than the lung tumor. COPD patients with a history of another lung disease such as asthma or interstitial lung disease were excluded.

This study was conducted according to national ethical and professional guidelines on the use human body material (“Code of conduct; Dutch federation of biomedical scientific societies”; https://www.federa.org/codes-conduct) and the Research Code of the University Medical Center Groningen (https://www.umcg.nl/EN/Research/Researchers/General/ResearchCode/Paginas/default.aspx).

Lung fibroblasts used in this study are derived from left-over lung material after lung surgery and transplant procedures. Currently, this material is not subject to the act on medical research involving human subjects in the Netherlands and therefore an ethics waiver was provided by the Medical Ethical Committee of the University Medical Center Groningen (METc UMCG). All samples and clinical information were de-identified before experiments were performed.

### Primary lung fibroblast culture

Primary parenchymal fibroblasts were isolated, cultured and stored in liquid nitrogen until further use as described previously [19]. The fibroblasts were cultured with Ham's F12 medium supplemented with 10% (v/v) fetal calf serum (FCS), 100 U/ml penicillin/streptomycin and 200 mM L-glutamine (all from Lonza, Breda, The Netherlands) at 37°C in a 90% (v/v) humidified atmosphere with 5% (v/v) CO_2_. The experiments were performed on these fibroblasts at passage 5. For the initial array analysis control fibroblasts in the discovery group were stimulated with 100 U/ml TGF-β1 (equivalent to 3.1 ng/ml) (R&D Systems, Abingdon, UK) in complete Ham's F12 medium containing 0.5% (v/v) FCS for 24 h. In the replication group, fibroblasts were stimulated with 2.5 ng/ml and 7.5 ng/ml TGF-β1 for 24 h.

### RNA isolation

Total RNA was isolated from primary parenchymal lung fibroblasts using TRIzol (Invitrogen, Carlsbad, CA, USA), according to the protocol of the manufacturer. RNA in the total (T) fraction of the immunoprecipitation of argonaute-2 (Ago2-IP) was isolated using miRNeasy Mini Kit (Qiagen, Venlo, The Netherlands), whereas RNA from the IP fraction was isolated using miRNeasy Micro Kit (Qiagen) according to manufacturer’s protocol. The RNA concentration was measured with a NanoDrop 1000 Spectrophotometer (Thermo Scientific, Wilmington, DE, USA).

### MiRNA expression profiling

RNA samples were hybridized with the Human GeneChip miRNA 1.0 array (Affymetrix), containing 847 probe sets for human miRNAs. Robust Multi-array Average was performed using GeneSpring GX version 13.1.1 software (Agilent Technologies, Santa Clara, CA, USA) for the quantile normalization of the probe-level intensity measurements. MiRNAs were filtered based on the following criteria: expression values in the range of 75^th^-100^th^ percentile in at least 50% of all samples (9 out of 18). After filtering, 205 miRNAs were left for further analyses. Statistical analyses in GeneSpring GX software for miRNA expression profiling were performed using the paired samples t-test and Benjamini-Hochberg false discovery rate (FDR) to correct for multiple testing. An FDR of p<0.05 was considered statistically significant. Unsupervised hierarchical clustering with Pearson's correlation was performed to generate a heatmap of differentially expressed miRNAs using Genesis software version 1.7.6 (Graz University of Technology, Graz, Austria) [20]. All differentially expressed miRNAs with a fold change (FC) ≥1.5 and a normalized signal intensity value of at least 75 in unstimulated or TGF-β1-stimulated fibroblasts were selected for validation.

### cDNA syntheses and qRT-PCR for ECM genes and α-SMA

cDNA was synthesized from 100 ng total RNA using random primers and Superscript II (all from Invitrogen). To check whether the TGF-β1 stimulation was successful, expression of several TGF-β1-inducible ECM genes (*fibronectin-1* (*FN1*), *collagen type I alpha I* (*COL1A1*), and *alpha-smooth muscle actin* (*α-SMA*) [1, 2]), was tested by qRT-PCR using the LightCycler®480 Real-Time PCR System (Roche Diagnostics GmbH, Mannheim, Germany). TaqMan Gene Expression Assays (FN1: Hs00365052_m1, COL1A1: Hs00164004_m1, α-SMA: Hs00426835_g1, RPS9: Hs02339424_g1 (all from Life Technologies, Bleiswijk, The Netherlands)), primers and probe (RP2: forward primer 5’-CGTACGCACCACGTCCAAT-3’, reverse primer 5’-CAAGAGAGCCAAGTGTCGGTAA-3’, probe 5’-TACCACGTCATCTCCTTTGATGGCTCCTAT-3’), primers (18S: forward primer 5’-CGGCTACCACATCCAAGGA-3’, reverse primer 5’-CCAATTACAGGGCCTCGAAA-3’), and qPCR MasterMix Plus (Eurogentec, Liege, Belgium) or SYBR green PCR master mix (Applied Biosystems, Carlsbad, CA, USA) were used. *Ribosomal protein S9* (*RPS9*) (in the discovery group), and *18S rRNA* (*18S*) and *RNA polymerase II* (*RP2*) (in the replication group) were used as reference genes. Samples including a no template control as a negative control were run in triplicate and the formula 2^-ΔCp^ was used to calculate the relative mRNA expression levels.

### cDNA syntheses and qRT-PCR for miRNAs

In order to validate and replicate the expression of the selected miRNAs, qRT-PCR was performed. Briefly, 10 ng of total RNA was first reverse transcribed in a multiplexed manner using reverse transcription primers from TaqMan^**®**^ microRNA Assay kits (RNU48 (Assay ID: 001006), hsa-miR-455-3p (Assay ID: 002244), hsa-miR-490-3p (Assay ID: 001037), hsa-miR-490-5p (Assay ID: 241012_mat), hsa-miR-21-3p (Assay ID: 002438), hsa-miR-143-3p (Assay ID: 000466); Applied Biosystems) as described previously [21]. A no template control was included for the cDNA synthesis to exclude primer dimers.

Next, qRT-PCR was performed in qPCR MasterMix Plus (Eurogentec) and TaqMan microRNA assay (Applied Biosystems). Small nucleolar RNA, C/D box 48 (RNU48) was used as reference gene. Samples including the no template control of both cDNA synthesis and qRT-PCR were run in triplicate and the formula 2^-ΔCp^ was used to calculate the relative miRNA expression levels. The data were analyzed using LightCycler®480 software release 1.5.0 (Roche Diagnostics GmbH).

### Immunoprecipitation of Ago2-RISC complex

To identify the miRNA-targetome of primary parenchymal lung fibroblasts of control subjects, Ago2-RIP-Chip was performed as described previously [22, 23]. We used primary parenchymal lung fibroblasts from two ex-smoking controls (control 1 and 2) with and without TGF-β1 (7.5 ng/ml) stimulation. In brief, 17–20 million cells were harvested and lysed using polysome lysis buffer. Cell lysates were incubated overnight with monoclonal mouse anti-human Ago2 (Clone 2E12-1C9, Abnova, Taipei City, Taiwan) coated Sepharose G beads (Abcam, Cambridge, UK). Lysates generated from the same number of cells were incubated with normal mouse polyclonal IgG_1_ isotype control (12–371, Merck Millipore, Amsterdam, The Netherlands) coated beads which served as a negative control for the IP procedure. To monitor the efficiency of the Ago2-IP, protein samples of the total (T), flow through (FT) and IP fractions were loaded on a 7.5% (w/v) polyacrylamide gel. Western blotting was performed as described previously [22].

### mRNA expression profiling and identification of the miRNA-targetomes

To determine which transcripts are enriched in the IP fraction after Ago2-IP, mRNA expression profiling was performed using G3 Human Gene Expression 8x60K v3 Microarrays (Agilent Technologies). RNA of the T and IP fraction (50–80 ng) was labeled with cyanine 3 (Cy3) and cyanine 5 (Cy5) using the Two-Color Low Input Quick Amp Labeling Kit (Agilent Technologies) according to manufacturer's protocol Version 6.9.1. NanoDrop 1000 Spectrophotometer (Thermo Scientific) was used to quantify the cRNA yield and to determine the specific activity.

Dye-swap hybridizations were performed overnight using Gene Expression Hybridization Kit (Agilent Technologies) and slides were scanned with the Agilent SureScan Microarray Scanner (Agilent Technologies) using the two-color gene expression protocol GE2_1200_Jun14. Feature Extraction software version 12.0.1.1 was used to extract information from probe features from our microarray scan data. The data was analyzed using GeneSpring GX version 13.1.1 software (Agilent Technologies). Only probes with consistent values in the dye-swap experiments (0.5>Cy3/Cy5<2) were used for further analyses. Out of the 58,341 probes on the microarray, 52,843 showed consistent values in the IP fraction and 51,588 in the T fraction of control 1, of which 47,917 were overlapping between the IP and T fractions. In the TGF-β1-stimulated fibroblasts of control 1, 51,991 probes in the IP fraction and 52,210 probes in the T fraction were consistent between the dyes of which 47,655 probes were overlapping between the fractions. For control 2, 50,963 and 49,118 probes were consistent for the IP and T fractions, respectively, and 44,551 probes were in overlap between the two fractions. In the TGF-β1-stimulated fibroblasts of control 2, 52,011 probes in the IP fraction and 50,654 probes in the T fraction were consistent between the dyes of which 46,404 probes were overlapping between the fractions. For each of the four IP-experiments, the list of probes with consistent signal intensities between the two dyes was further filtered using the flagged present status (detectable above background according to the feature extracting software). All probes flagged present in at least one out of two conditions (IP and T) were included in the further analyses (37–43% of probes discarded of the consistent list). Finally, the lists of the two unstimulated samples were overlapped (24,502 remaining) and similarly, the two TGF-β1-stimulated samples were overlapped (25,939 remaining). Both the IP/T ratios of the two unstimulated fibroblast samples and those of the two TGF-β1-stimulated fibroblasts samples were statistically correlated using the Pearson correlation test. The miRNA-targetomes were defined as the top 1,500 of the most IP-enriched transcripts in the fibroblast samples of both control subjects. To identify which of the miR-455-3p and miR-21-3p target genes are influenced by TGF-β1 stimulation, we ranked the Ago2-IP enriched and predicted targets by the IP/T ratio in each of the four IP experiments (Control 1 and 2, both with and without TGF-β1). For miR-455-3p that is broadly conserved, only the predicted targets with conserved binding sites were considered in these analyses (TargetScan version 7.1 [24]). MiR-21-3p is poorly conserved; therefore the predicted miR-21-3p targets with a cumulative weighted context^++^ score <-0.2 were included. The target genes that are affected by the TGF-β1-dependent modulation of miR-455-3p or miR-21-3p are those genes that are more prominently IP-enriched in TGF-β1-stimulated fibroblasts compared to unstimulated fibroblasts. This was defined according to the following criteria 1) predicted target gene is present in the top 1,500 most enriched transcripts in the TGF-β1-stimulated fibroblasts; 2) predicted target gene is more enriched in the TGF-β1-stimulated sample with a difference in ranking of ≥300; 3) the more pronounced enrichment in the TGF-β1-stimulated fibroblasts is consistent in both controls.

### Gene ontology and pathway analysis

Gene set enrichment analysis (GSEA software v2.2.2) was performed using the Molecular Signatures Database (MSigDB v5.1) to check for enrichment of miRNA target genes sets and other gene ontologies [25]. The probes were ranked based on the mean IP/T ratio of the two control subjects.

GeneNetwork analysis was performed to identify enrichment of biological processes and pathways (KEGG and reactome) amongst the TargetScan (version 7.1) predicted and Ago2-IP enriched targets of miR-455-3p and miR-21-3p [24]. GeneNetwork is based on an independent gene expression dataset and predicts (currently unknown) gene functions based on co-expression. This information is used to assign genes to specific pathways and biological processes [1, 26].

### Statistical analyses

IBM SPSS Statistics 22 software was used to compare differences in subject characteristics between control subjects and COPD patients, and between the two study groups using Mann-Whitney U test. Differences in ECM gene, α-SMA gene and miRNA expression after TGF-β1 stimulation were analyzed using one-sided paired Wilcoxon signed rank tests. The Mann-Whitney U test was used to compare expression of genes and miRNAs between controls and COPD patients. Chi-square test was used to test differences in the percentage of predicted targets in the Ago2-IP fraction for miR-455-3p and miR-21-3p in the miRNA-targetome compared to the percentage of predicted targets in all expressed genes. A p-value below 0.05 was considered statistically significant.

## Results

### Subject characteristics

The clinical characteristics of both study groups are shown in Table 1. The discovery group consisted of 4 ex- and 5 current smoking controls. The replication group consisted of 4 ex- and 6 current smoking controls and 15 COPD patients. Both age and number of pack-years did not differ between both study groups, or between controls and COPD patients. Furthermore, the Forced Expiratory Volume in one second (FEV_1_) and the Forced Expiratory Volume in one second/Forced Vital Capacity (FEV_1_/FVC) ratio in controls did not differ between discovery and replication groups.

**Table 1:** Subject characteristics.

Subjects	Discovery group	Replication group	
	Control subjects^d^	Control subjects^d^	COPD patients
N	9	10	15
Male/Female, n	0/9	3/7	7/8
Age, years^a^	55 (48.0–60.5)	63.0 (48.5–69.0)	59.0 (52.0–71.0)
FEV_1_, % predicted^a^^,^^b^	89.9 (82.8–100.6)	92.4 (90.4–98.4)	22.3 (16.8–39.3)
FEV_1_/FVC, %^a^^,^^c^	73.6 (70.9–81.9)	74.8 (73.0–80.3)	28.7 (24.7–43.5)
Stage II/III/IV COPD, n	–	–	2/3/10
Ex-/current smoker, n	4/5	4/6	15/0
Pack-years, n^a^	33.0 (21.5–39.0)	33.0 (25.5–51.8)	40.0 (26.3–55.0)

^a^ Median (Interquartile range).

^b^ FEV_1_, % predicted, percentage of Forced Expiratory Volume in one second of the predicted normal value for an individual of the same sex, age and height.

^c^ FEV_1_/FVC, Forced Expiratory Volume in one second/Forced Vital Capacity ratio expressed in percentage, a measurement for obstruction/restriction in the lungs.

^d^ Three control subjects were overlapping in both study groups.

### Differentially expressed miRNAs after TGF-β1 stimulation

**Fig 1:**
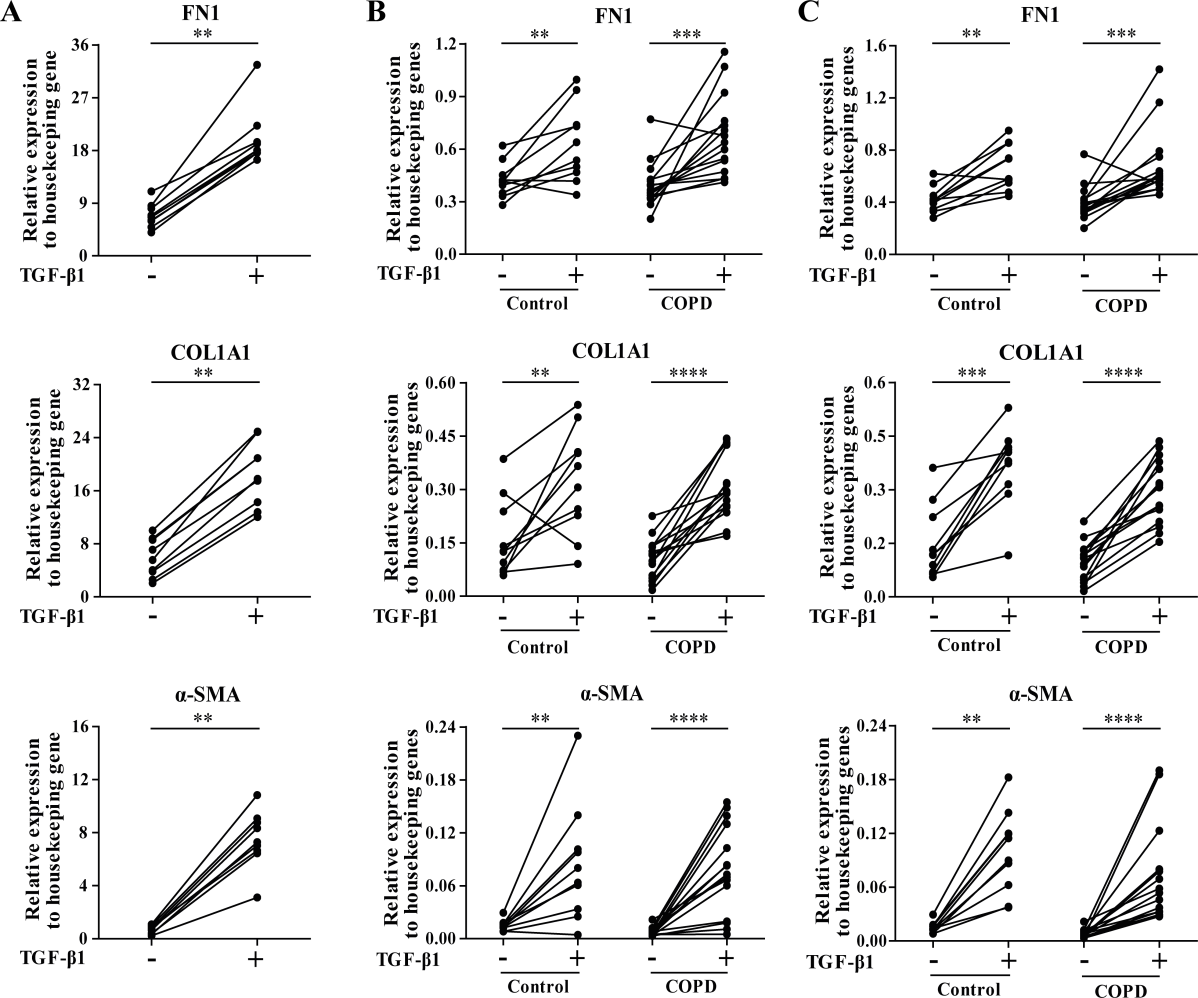
Upregulation of ECM genes and α-SMA after TGF-β1 stimulation in primary parenchymal lung fibroblasts. (A) Effective TGF-β1 stimulation of control fibroblasts in the discovery group was confirmed by the upregulation of *FN1* (fibronectin 1), *COL1A1* (collagen type I alpha I) and *α-SMA* (alpha-smooth muscle actin), genes that are well-known to be affected by TGF-β. (B) These genes were also upregulated in the control and COPD fibroblasts in the replication group after 2.5 ng/ml TGF-β1 and (C) after 7.5 ng/ml TGF-β1 stimulation. Data are presented as relative expression (2^-ΔCp^). **p<0.01, ***p<0.001, ****p<0.0001.

**Fig 2:**
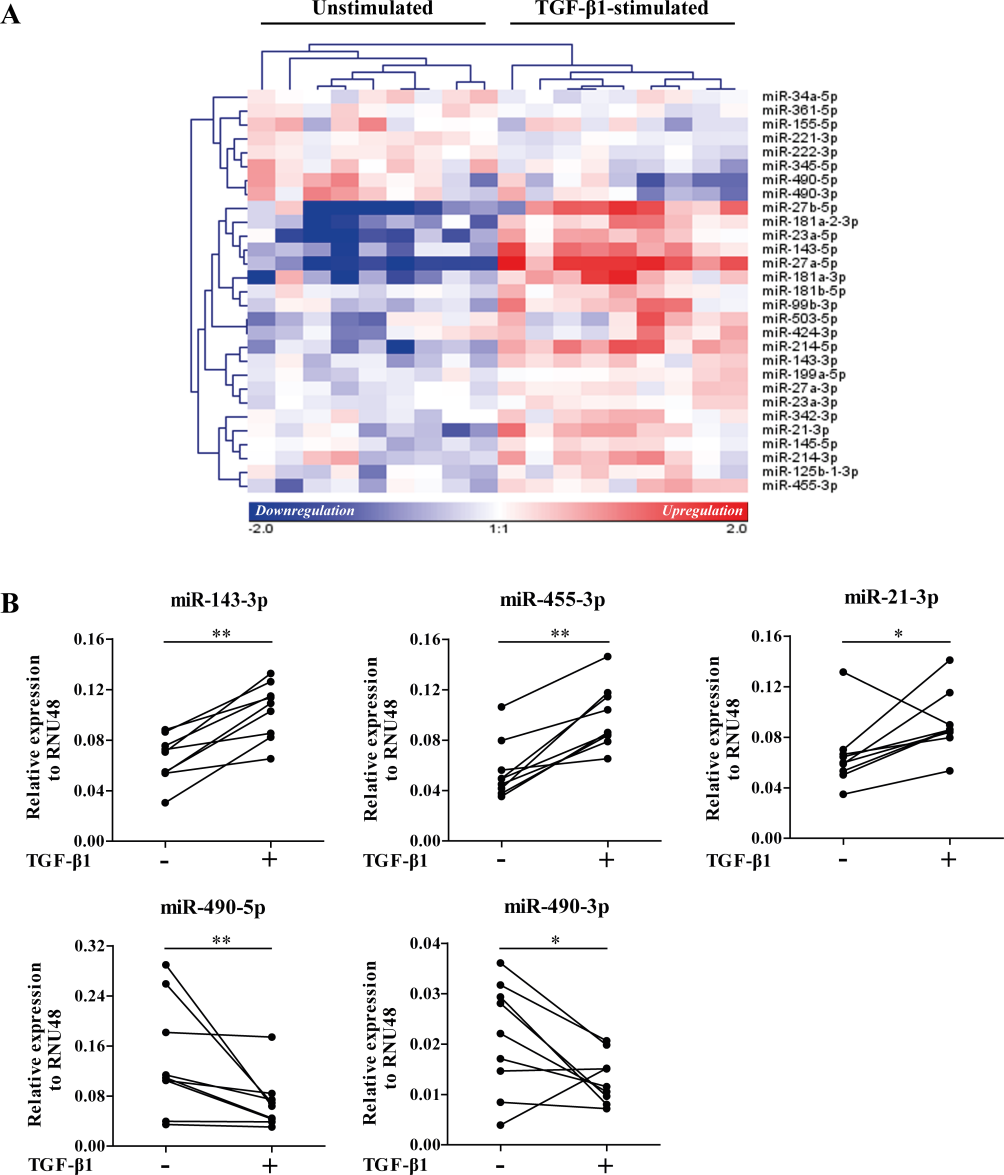
Differentially expressed miRNAs after TGF-β1 stimulation in control lung fibroblasts in the discovery group. (A) The miRNA expression in primary parenchymal lung fibroblasts of control subjects with and without TGF-β1 stimulation was determined by microarray. Unsupervised hierarchical clustering was used to generate the heatmap and pearson correlation was used as the distance metric. Twenty-nine miRNAs were differentially expressed after TGF-β1 stimulation (FDR<0.05). The heatmap shows the median-centered expression of the 29 miRNAs of which 8 miRNAs were downregulated and 21 miRNAs were upregulated after TGF-β1 stimulation. (B) Validation of differentially expressed miRNAs after TGF-β1 stimulation in the discovery group using qRT-PCR. Data are presented as relative expression (2^-ΔCp^) normalized to RNU48. *p<0.05, **p<0.01.

In the discovery group, TGF-β1 stimulation caused a significant induction of the known TGF-β-regulated genes *FN1*, *COL1A1*, and *α-SMA* (p<0.01, Fig 1A) confirming successful TGF-β1 stimulation. We found 29 miRNAs to be differentially expressed after TGF-β1 stimulation in the discovery group (FDR<0.05, Fig 2A). Out of these 29 miRNAs, 8 miRNAs were downregulated and 21 miRNAs were upregulated. Fourteen out of 29 miRNAs had a fold change of at least 1.5, of which 12 showed increased expression after TGF-β1 stimulation (Table 2). Based on the signal intensity, 6 of these 14 miRNAs were selected for qRT-PCR validation and replication (marked in bold in Table 2).

**Table 2:** List of miRNAs with at least 1.5 fold change after TGF-β1 stimulation.

miRNA	FC	Control subjects			COPD patients		
		Signal intensity -/+ TGF-β1	Validated	Replicated	Replicated		
**miR-143-3p**	**1.5**	**1098/1617**	**Yes**	**No**	**Yes**		
**miR-503-5p**	**1.6**	**244/384**	**BDL**^**a**^	**BDL**^**a**^	**BDL**^**a**^		
**miR-455-3p**	**1.6**	**214/343**	**Yes**	**Yes**	**Yes**		
**miR-21-3p**	**1.8**	**87/157**	**Yes**	**Yes**	**Yes**		
miR-23a-5p	3.5	20/69					
miR-143-5p	3.1	21/64					
miR-214-5p	3.0	19/56					
miR-27a-5p	8.5	6/53					
miR-181a-2-3p	2.7	18/49					
miR-99b-3p	1.9	23/43					
miR-27b-5p	5.6	7/39					
miR-181a-3p	3.5	11/37					
**miR-490-5p**	**-1.6**	**182/111**	**Yes**	**No**	**No**		
**miR-490-3p**	**-1.5**	**98/65**	**Yes**	**No**	**No**		

^a^ BDL, Below detection level.

We validated the TGF-β1-induced expression changes of five out of six miRNAs (miR-143-3p, miR-455-3p, miR-21-3p, miR-490-5p and miR-490-3p; p<0.05) in our discovery group using qRT-PCR (Fig 2B). For miR-503-3p the expression was below the detection limit of the assay.

### Replication of TGF-β1 effects on miRNA expression in COPD and control fibroblasts

In the replication group, we included primary parenchymal lung fibroblasts from control subjects as well as from COPD patients. Again, the TGF-β1 stimulation of 2.5 ng/ml (p<0.01, Fig 1B) and 7.5 ng/ml was successful as it significantly induced the mRNA expression of *FN1*, *COL1A1*, and *α-SMA* in the primary parenchymal lung fibroblasts of both controls and COPD patients (p<0.01, Fig 1C).

**Fig 3:**
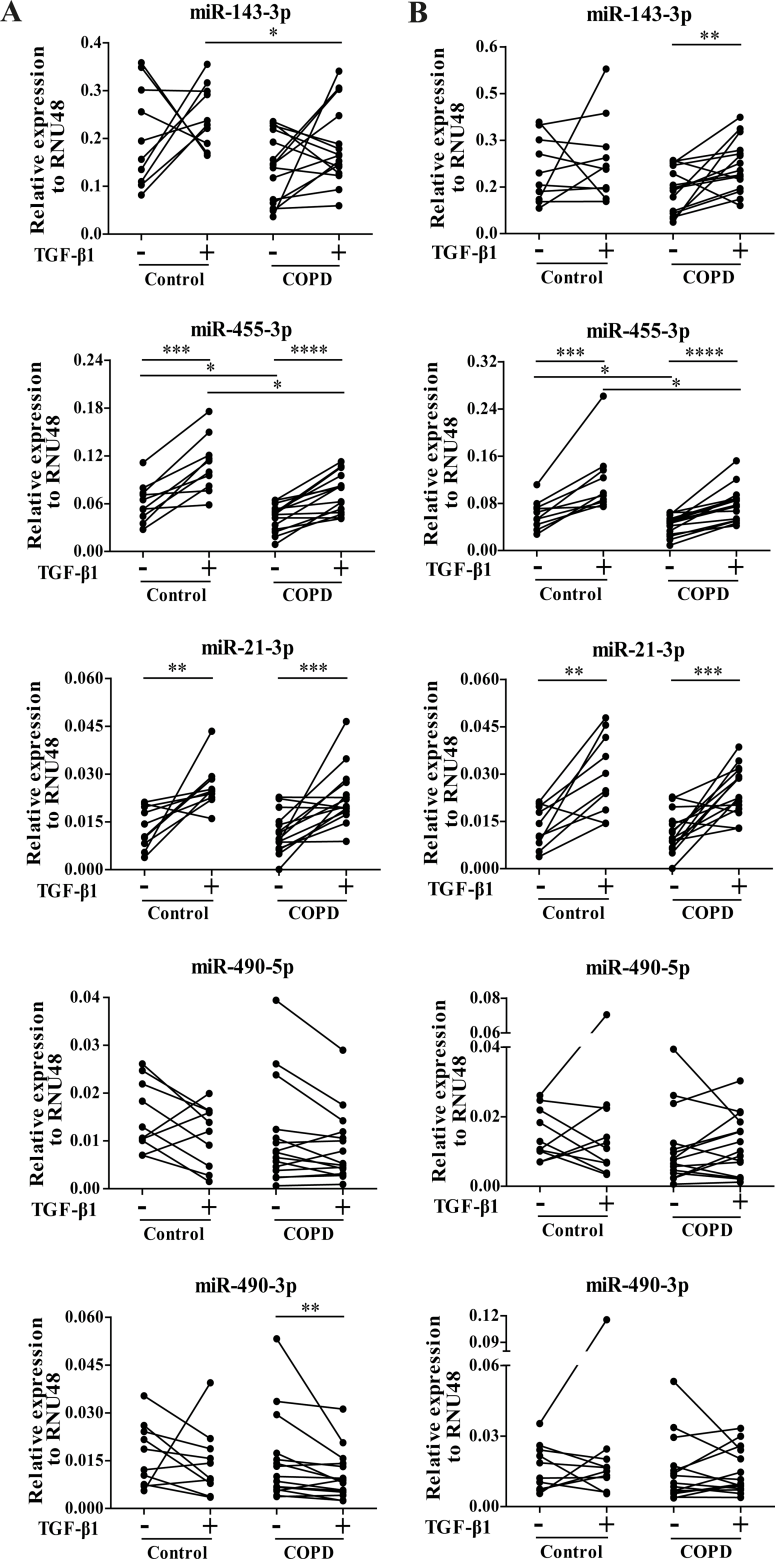
Replication of differentially expressed miRNAs after TGF-β1 stimulation using qRT-PCR. To replicate the TGF-β1-induced expression changes of the validated miRNAs, qRT-PCR was performed on the control and COPD fibroblasts in the replication group (A) stimulated with 2.5 ng/ml TGF-β1 and (B) stimulated with 7.5 ng/ml TGF-β1. Data are presented as relative expression (2^-ΔCp^) normalized to RNU48. *p<0.05, **p<0.01, ***p<0.001, ****p<0.0001.

In the primary lung fibroblasts of control subjects, we were able to replicate the expression changes of 2 out of 5 miRNAs (miR-455-3p and miR-21-3p) after 2.5 ng/ml TGF-β1 (p<0.01, Fig 3A) as well as after 7.5 ng/ml TGF-β1 stimulation (p<0.01, Fig 3B). In the fibroblasts of COPD patients, we found significant upregulation of miR-455-3p and miR-21-3p after 2.5 ng/ml as well as after 7.5 ng/ml TGF-β1 (p<0.0001). In addition, we found a significant downregulation of miR-490-3p in COPD fibroblasts after stimulation with 2.5 ng/ml (p<0.01, Fig 3A) and a significant induction of miR-143-3p in COPD fibroblasts after stimulation with 7.5 ng/ml TGF-β1 (p<0.01, Fig 3B), which both were not observed in the replication of the control subjects.

As a secondary aim we also compared the expression levels of the 5 selected miRNAs in primary parenchymal lung fibroblasts between control subjects and COPD patients. MiR-455-3p levels were significantly lower in COPD patients (p<0.05) both at basal levels and after TGF-β1 stimulation (p<0.05, Fig 3A and 3B). In addition, a lower miR-143-3p expression was found in the 2.5 ng/ml TGF-β1-stimulated lung fibroblasts of COPD patients compared to those of control subjects (p<0.05, Fig 3A).

### Identification of the miRNA-targetomes in primary lung fibroblasts

**Fig 4:**
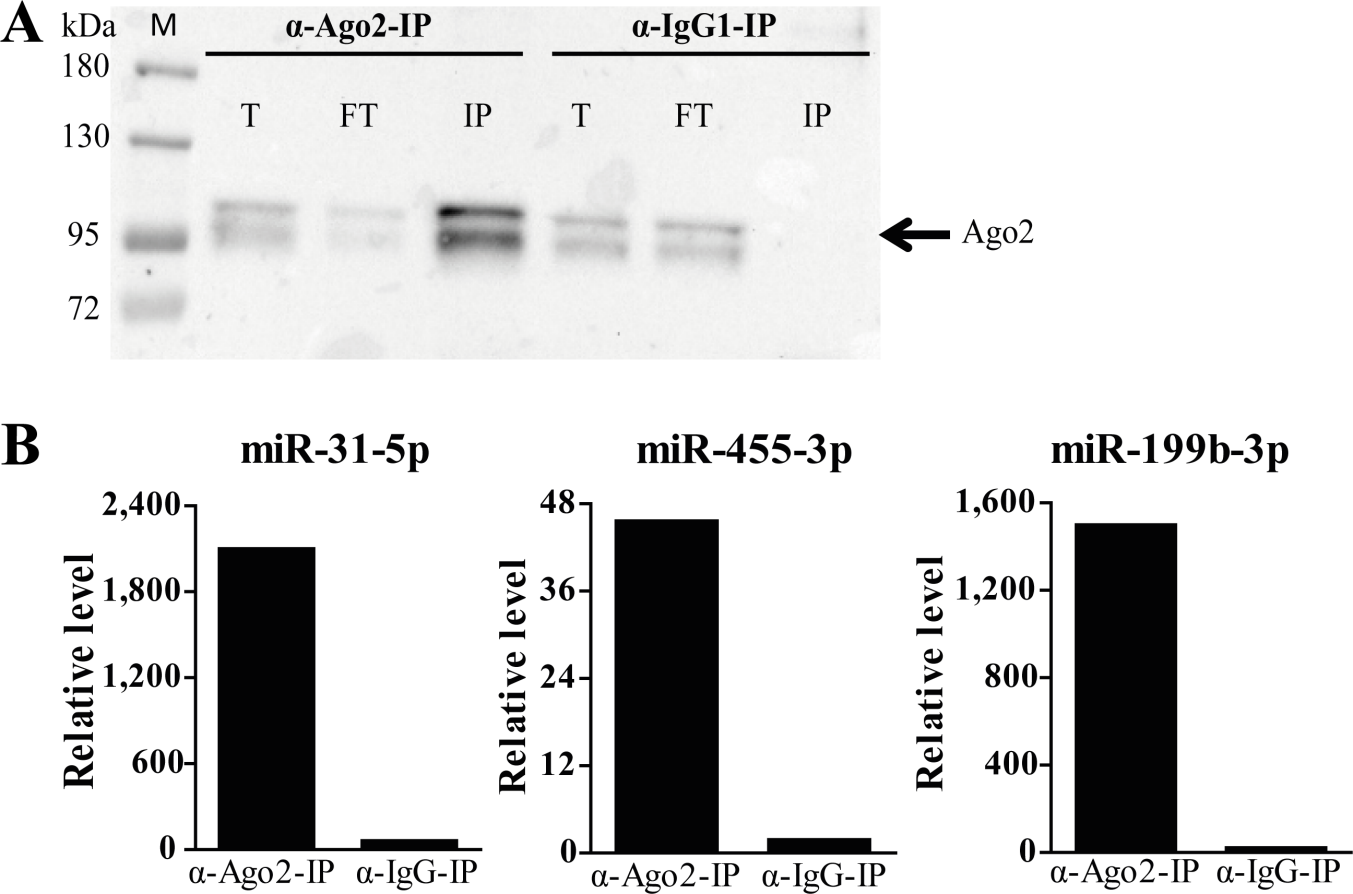
Efficiency of Ago2-immunoprecipitation. (A) Western blot of Ago2 protein in the Ago2- and IgG-immunoprecipitation. M marker; T Total fraction; FT Flow through fraction; IP Immunoprecipitation fraction. Arrow indicates the Ago2 protein. Ago2 protein can be detected in the Ago2-IP fraction, while it cannot be detected in the IgG_1_-IP fraction. (B) To confirm miRNA enrichment in the IP fraction, qRT-PCR was performed for three randomly selected miRNAs expressed in lung fibroblasts, i.e. miR-31-5p, miR-455-3p and miR-199b. The levels of these miRNAs are strongly increased in the Ago2-IP fraction compared to the IgG_1_-IP fraction.

To define the mRNAs that are actively targeted by miRNAs in primary parenchymal lung fibroblasts we profiled the mRNAs recovered from Ago2-IP from unstimulated and TGF-β1-stimulated primary parenchymal lung fibroblasts of two control subjects. The efficiency of the IP procedure was confirmed by Western blotting of Ago2, showing a clear enrichment of the Ago2 protein in the IP fraction of the Ago2-IP sample and not in the IgG1 control (Fig 4A). Additional qRT-PCR of three miRNAs also showed a clear increase in miRNA levels in the Ago2-IP fractions (Fig 4B). The Ago2-IP results were consistent between the two controls, as the IP/T ratios of the 24,502 probes corresponding to 17,129 unique genes in the unstimulated lung fibroblasts and the 25,939 probes corresponding to 18,037 unique genes in the TGF-β1-stimulated lung fibroblasts were highly correlated between the two subjects (R^2^ = 0.8164, p<0.0001 and R^2^ = 0.7257, p<0.0001, respectively). GSEA analysis using the ranking of the IP/T ratio of all genes that are consistently expressed in unstimulated lung fibroblasts of the two subjects demonstrated an enrichment of 17 miRNA-target gene sets in the top 30 enriched gene sets (Table 3). The top 5 most significant miRNA gene sets that are identified in primary lung fibroblasts were the miRNA (seed families of) Let-7, miR-29, miR-26, miR-181 and miR-101. Similar GSEA analysis was also performed on all consistently expressed genes in the TGF-β1-stimulated lung fibroblasts of the two subjects and showed 15 miRNA-target gene sets in the top 30 enriched gene sets (Table 3). In these TGF-β1-stimulated lung fibroblasts, the most significant active miRNA gene sets were the miRNA (seed families of) Let-7, miR-26, miR-202, miR-27 and miR-29. Together these analyses support the robustness of our IP approach.

**Table 3:** Top 30 most enriched gene sets in unstimulated and TGF-β1-stimulated lung fibroblasts.

	Unstimulated		TGF-β1-stimulated	
Gene sets	Rank	FDR	Rank	FDR
CTACCTC,LET-7A,LET-7B,LET-7C,LET-7D,LET-7E,LET-7F,MIR-98,LET-7G, LET-7I^a^	1	0.0	2	0.0
CHR19P12	2	0.0	1	0.0
TGGTGCT,MIR-29A,MIR-29B,MIR-29C^a^	3	0.0	11	0.0
TACTTGA,MIR-26A,MIR-26B^a^	4	0.0	6	0.0
GENTILE_UV_HIGH_DOSE_DN	5	0.0	4	0.0
TGAATGT,MIR-181A,MIR-181B,MIR-181C,MIR-181D^a^	6	0.0	12	0.0
GTACTGT,MIR-101^a^	7	0.0	35	9.46x10^-5^
ATAAGCT,MIR-21^a^	8	0.0	19	0.0
BONCI_TARGETS_OF_MIR15A_AND_MIR16_1^a^	9	0.0	14	0.0
ZWANG_CLASS_3_TRANSIENTLY_INDUCED_BY_EGF	10	0.0	5	0.0
ACTACCT,MIR-196A,MIR-196B^a^	11	0.0	27	4.06x10^-5^
ATGTAGC,MIR-221,MIR-222^a^	12	0.0	22	4.98x10^-5^
WINZEN_DEGRADED_VIA_KHSRP	13	0.0	3	0.0
CTCAGGG,MIR-125B,MIR-125A^a^	14	0.0	21	5.21x10^-5^
DAZARD_RESPONSE_TO_UV_SCC_DN	15	0.0	26	4.21x10^-5^
ZWANG_DOWN_BY_2ND_EGF_PULSE	16	0.0	9	0.0
REACTOME_GENERIC_TRANSCRIPTION_PATHWAY	17	0.0	7	0.0
GTGCAAT,MIR-25,MIR-32,MIR-92,MIR-363,MIR-367^a^	18	0.0	18	0.0
GABRIELY_MIR21_TARGETS^a^	19	0.0	31	3.53x10^-5^
ATACCTC,MIR-202^a^	20	0.0	8	0.0
GENTILE_UV_RESPONSE_CLUSTER_D2	21	0.0	37	8.95x10^-5^
STK33_SKM_UP	22	0.0	16	0.0
CHEN_HOXA5_TARGETS_9HR_UP	23	0.0	34	6.46x10^-5^
GCACTTT,MIR-17-5P,MIR-20A,MIR-106A,MIR-106B,MIR-20B,MIR-519D^a^	24	4.58x10^-5^	20	0.0
ACTGTGA,MIR-27A,MIR-27B^a^	25	4.39x10^-5^	10	0.0
ACACTAC,MIR-142-3P^a^	26	4.22x10^-5^	54	3.69x10^-4^
GSE9988_ANTI_TREM1_VS_VEHICLE_TREATED_MONOCYTES_UP	27	4.07x10^-5^	24	4.56x10^-5^
STK33_UP	28	3.92x10^-5^	23	4.76x10^-5^
ATACTGT,MIR-144^a^	29	3.79x10^-5^	74	7.92x10^-4^
GSE9988_ANTI_TREM1_AND_LPS_VS_CTRL_TREATED_MONOCYTES_UP	30	3.66x10^-5^	17	0.0
GSE9988_ANTI_TREM1_VS_CTRL_TREATED_MONOCYTES_UP	32	3.43x10^-5^	13	0.0
AATGTGA,MIR-23A,MIR-23B^a^	47	2.09x10^-4^	15	0.0
AGCACTT,MIR-93,MIR-302A,MIR-302B,MIR-302C,MIR-302D,MIR-372,MIR-373,MIR-20E, MIR-520A,MIR-526B,MIR-520B,MIR-520C,MIR-520D^a^	31	3.54x10^-5^	25	4.38x10^-5^
GSE9988_ANTI_TREM1_AND_LPS_VS_VEHICLE_TREATED_MONOCYTES_UP	42	1.30x10^-4^	28	3.91x10^-5^
NAGASHIMA_EGF_SIGNALING_UP	105	1.40x10^-3^	29	3.78x10^-5^
NAGASHIMA_NRG1_SIGNALING_UP	65	3.70x10^-4^	30	3.65x10^-5^

^a^ MiRNA-target gene sets

**Fig 5:**
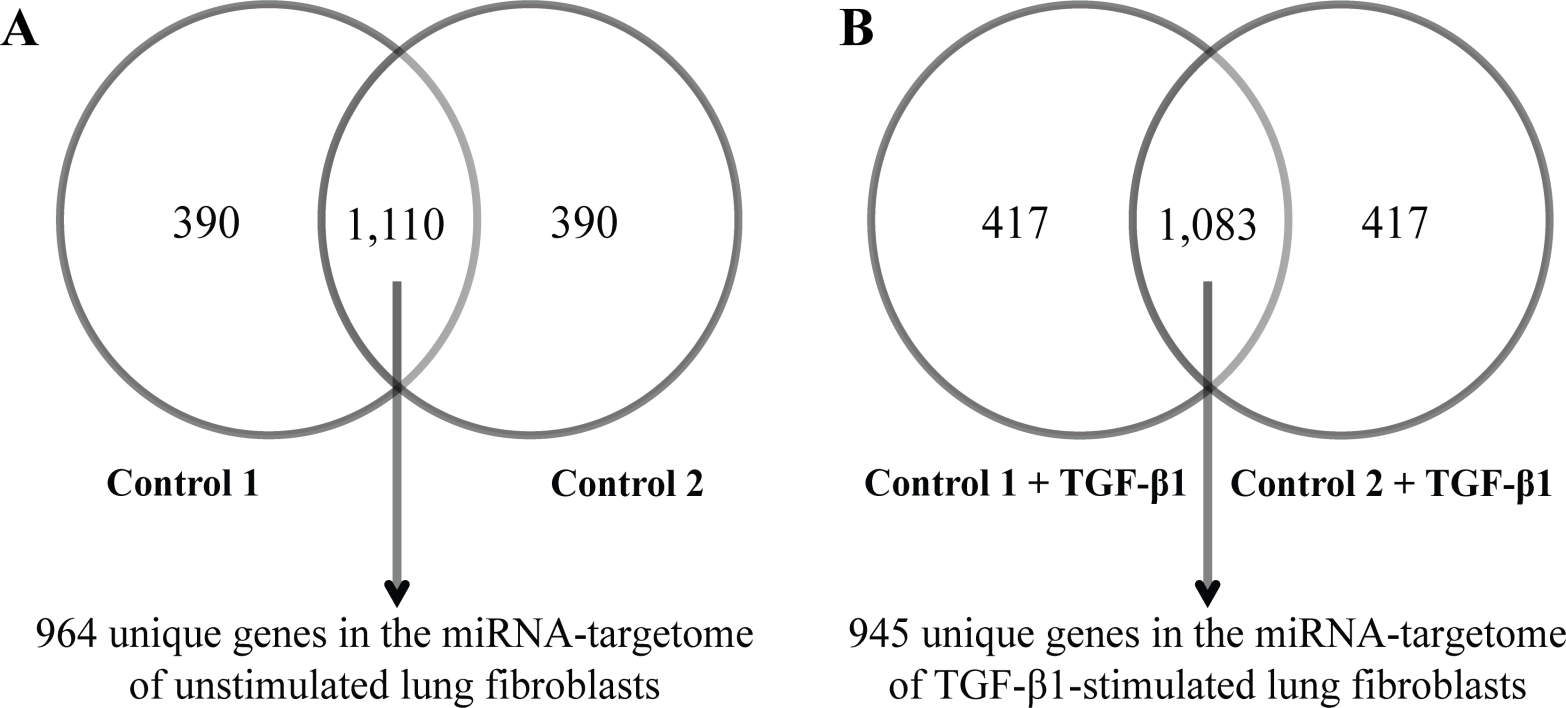
Defining the miRNA-targetomes of unstimulated and TGF-β1-stimulated lung fibroblasts. The overlap of the top 1,500 most IP-enriched probes in the (A) unstimulated and (B) TGF-β1-stimulated fibroblasts of the two control subjects are defined as the miRNA-targetome. The identified genes in the miRNA-targetomes are listed in S1 Tables.

The top 1,500 most IP-enriched probes that were consistently enriched in the fibroblasts of the two control subjects are defined as the parenchymal lung fibroblast “miRNA-targetome”. Of the top 1,500 most IP-enriched probes in the unstimulated primary parenchymal lung fibroblasts of the two control subjects, we identified 1,110 probes overlapping between the two control subjects, which corresponded to 964 unique genes, and these were defined as the miRNA-targetome of unstimulated cells (Fig 5A). Of the top 1,500 most IP-enriched probes in the TGF-β1-stimulated lung fibroblasts of the two control subjects, we identified 1,083 probes overlapping between the control subjects, which corresponded to 945 unique genes, and these were identified as the miRNA-targetome of the TGF-β1-stimulated cells (Fig 5B). The IP/T ratios of the top 1,500 most IP-enriched probes in the unstimulated and TGF-β1-stimulated lung fibroblasts of the two control subjects and the identified genes in the miRNA-targetomes are shown in S1 Tables.

### MiR-455-3p and miR-21-3p predicted targets are enriched in the lung fibroblast targetomes

As the TGF-β1 effect on miR-455-3p and miR-21-3p was consistent throughout the experiments, we decided to focus on these two miRNAs. We determined which transcripts might be affected by the TGF-β1-induced changes in miR-455-3p and miR-21-3p expression by identifying TargetScan predicted targets of these two miRNAs within the miRNA-targetome lists.

**Fig 6:**
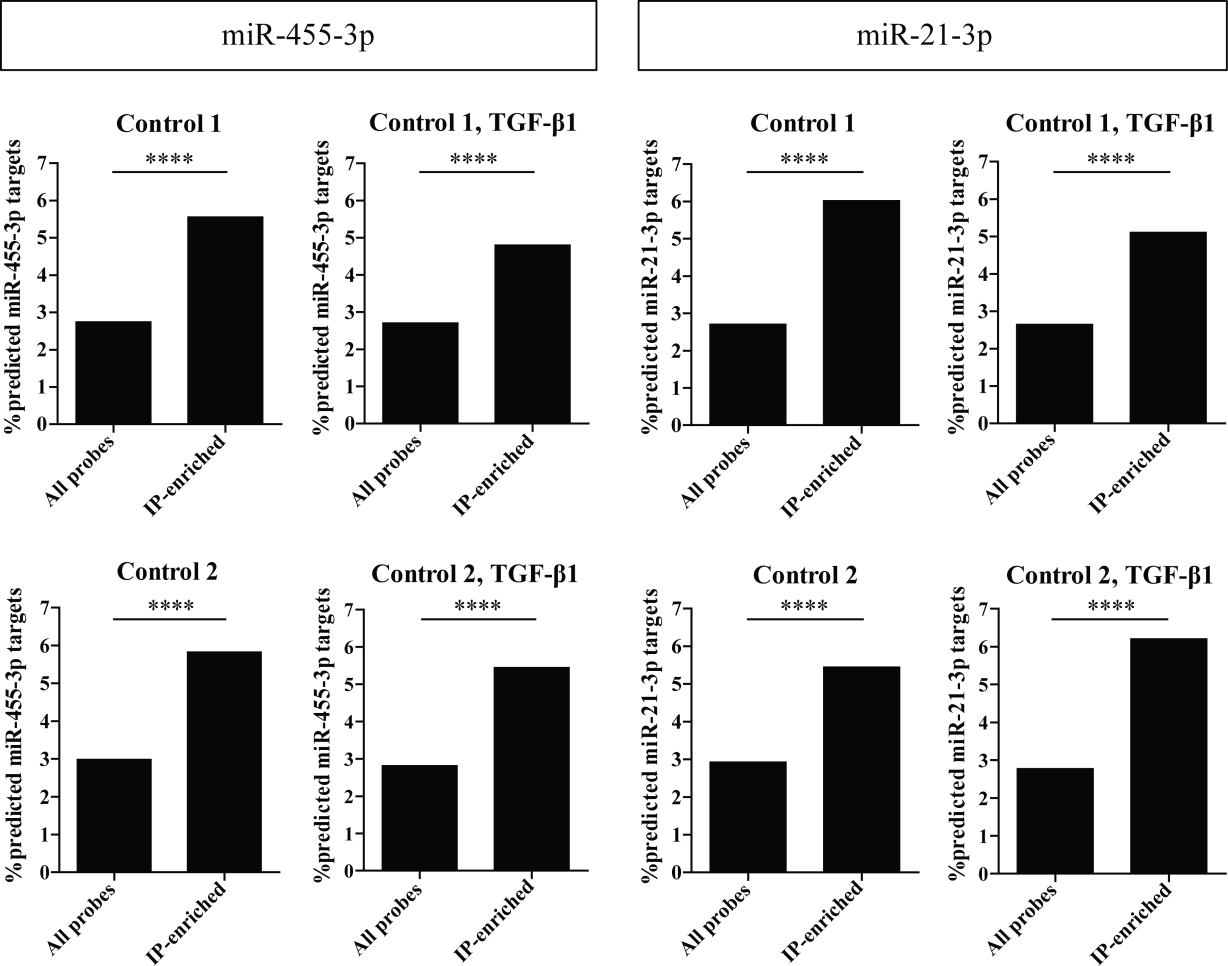
Significant Ago2-IP-enrichment of miR-455-3p and miR-21-3p predicted targets. For each miRNA, the percentages of predicted targets were calculated in all expressed genes and in the top 1,500 most IP-enriched genes in all four IP experiments. Chi-square test was used to determine whether the number of predicted targets in the Ago2-IP fraction for miR-455-3p and miR-21-3p in the top 1,500 most enriched genes was significant different from the expected based on the number of predicted targets in all expressed genes.

In the unstimulated as well as in the TGF-β1-stimulated fibroblasts of both subjects, a significant increase of genes with at least one conserved miR-455-3p binding site was observed in the top 1,500 most IP-enriched genes, compared to the predicted miR-455-3p targets amongst all expressed genes (p<0.0001, Fig 6). In total, 57 and 50 predicted miR-455-3p target genes were enriched in the miRNA-targetome of unstimulated and TGF-β1-stimulated lung fibroblasts, respectively.

Also for miR-21-3p a significant increase in target genes was observed in the top 1,500 most enriched genes (p<0.0001, Fig 6). For miR-21-3p, 55 and 54 predicted target genes were enriched in the miRNA-targetome of unstimulated and TGF-β1-stimulated lung fibroblasts, respectively.

### Identification of the processes and pathways of the miR-455-3p and miR-21-3p target genes

To investigate the possible functions of miR-455-3p and miR-21-3p in the unstimulated and TGF-β1-stimulated lung fibroblasts, we subjected the predicted and IP-enriched targets of these miRNAs to GeneNetwork analysis, focusing on Biological processes, Kegg pathways and Reactome gene sets. Per collection, the top 10 most significant biological processes and pathways of the 57 and 50 predicted miR-455-3p targets of unstimulated and TGF-β1-stimulated fibroblasts are depicted in Table 4. Several biological processes and pathways are related to very general processes such as gene expression, differentiation, proliferation, various types of cancer and signaling pathways. Interestingly, the TGF-β signaling pathway (p≤5x10^-6^) and the Wnt signaling pathway (p≤7x10^-6^) were significantly enriched among these genes both in the unstimulated and in the TGF-β1-stimulated lung fibroblasts. Furthermore, the TGF-β receptor signaling pathway was significantly enriched in the TGF-β1-stimulated fibroblasts (p = 2x10^-8^). Several of the predicted miR-455-3p targets were allocated to the TGF-β-related processes and pathway and to the Wnt signaling pathway by GeneNetwork as annotated or unannotated genes (Table 5).

**Table 4:** Most enriched biological processes/pathways within the Ago2-IP enriched and TargetScan predicted miR-455-3p target genes.

		Pathway/term	p-value
**No TGF-β1**	**Biological process**	Posttranscriptional regulation of gene expression	9 x 10^−10^
		Negative regulation of transcription from RNA polymerase II promoter	2 x 10^−9^
		Stress-activated protein kinase signaling cascade	5 x 10^−9^
		Regulation of protein serine/threonine kinase activity	9 x 10^−9^
		Intracellular receptor mediated signaling pathway	3 x 10^−8^
		Negative regulation of protein serine/threonine kinase activity	3 x 10^−8^
		Intracellular steroid hormone receptor signaling pathway	4 x 10^−8^
		Myeloid cell differentiation	5 x 10^−8^
		Negative regulation of cell proliferation	7 x 10^−8^
		Regulation of myeloid cell differentiation	7 x 10^−8^
	**KEGG**	Prostate cancer	2 x 10^−6^
		Adherens junction	3 x 10^−6^
		Pathways in cancer	3 x 10^−6^
		TGF-beta signaling pathway	4 x 10^−6^
		T cell receptor signaling pathway	5 x 10^−6^
		Wnt signaling pathway	7 x 10^−6^
		Colorectal cancer	9 x 10^−6^
		Neurotrophin signaling pathway	1 x 10^−5^
		Chronic myeloid leukemia	4 x 10^−5^
		ErbB signaling pathway	4 x 10^−5^
	**Reactome**	MAP kinase activation in TLR cascade	2 x 10^−5^
		Generic Transcription Pathway	5 x 10^−5^
		Transcriptional Regulation of White Adipocyte Differentiation	6 x 10^−5^
		Circadian Clock	7 x 10^−5^
		GAB1 signalosome	1 x 10^−4^
		MAPK targets/ Nuclear events mediated by MAP kinases	1 x 10^−4^
		PI3K/AKT activation	3 x 10^−4^
		TRAF6 mediated IRF7 activation	3 x 10^−4^
		Regulation of Lipid Metabolism by Peroxisome proliferator-activated receptor alpha	4 x 10^−4^
		NFkB and MAP kinases activation mediated by TLR4 signaling repertoire	4 x 10^−4^
		**Pathway/term**	**p-value**
**TGF-β1**	**Biological process**	Transforming growth factor beta receptor signaling pathway	2 x 10^−8^
		Posttranscriptional regulation of gene expression	5 x 10^−8^
		In utero embryonic development	9 x 10^−8^
		Regulation of myeloid cell differentiation	1 x 10^−7^
		Negative regulation of transcription from RNA polymerase II promoter	2 x 10^−7^
		Intracellular steroid hormone receptor signaling pathway	2 x 10^−7^
		Chordate embryonic development	2 x 10^−7^
		Embryo development ending in birth or egg hatching	3 x 10^−7^
		Androgen receptor signaling pathway	3 x 10^−7^
		Protein dephosphorylation	3 x 10^−7^
	**KEGG**	Pathways in cancer	4 x 10^−7^
		Colorectal cancer	7 x 10^−7^
		Prostate cancer	9 x 10^−7^
		Wnt signaling pathway	2 x 10^−6^
		Adherens junction	4 x 10^−6^
		Chronic myeloid leukemia	5 x 10^−6^
		TGF-beta signaling pathway	5 x 10^−6^
		Neurotrophin signaling pathway	2 x 10^−5^
		Small cell lung cancer	3 x 10^−5^
		Acute myeloid leukemia	4 x 10^−5^
	**Reactome**	Transcriptional Regulation of White Adipocyte Differentiation	3 x 10^−5^
		MAP kinase activation in TLR cascade	3 x 10^−4^
		Signaling by BMP	3 x 10^−4^
		Signaling by EGFR	4 x 10^−4^
		Signaling by Notch	5 x 10^−4^
		Signaling by EGFR in Cancer	6 x 10^−4^
		MAPK targets/ Nuclear events mediated by MAP kinases	9 x 10^−4^
		Metabolism of amino acids and derivatives	10 x 10^−4^
		Signaling by NODAL	10 x 10^−4^
		Regulation of Lipid Metabolism by Peroxisome proliferator-activated receptor alpha	10 x 10^−4^

**Table 5:** Predicted miR-455-3p and miR-21-3p targets present in miRNA-targetomes involved in TGF-β-related processes and pathway and Wnt signaling pathway.

miRNA	Lung fibroblasts	Process/Pathway	Annotated genes^a^	Unannotated genes^b^			
**miR-455-3p**	**Unstimulated**	TGF-beta signaling pathway (KEGG)	*ACVR2B*	*ATXN1*	*PPP2R2A*	*ZFHX4*	
				*BTG2*	*RUNX1*	*ZFP36L1*	
				*GATA6*	*TCF7L1*	*ZSWIM6*	
				*NPEPPS*	*TMEM64*		
				*PLAGL2*	*ZFHX3*		
		Wnt signaling pathway (KEGG)	*TCF7L1*	*ACTR10*	*GATA6*	*RARB*	
				*ACVR2B*	*NPEPPS*	*RCC2*	
				*ARPC2*	*POU2F1*	*SPRY1*	
				*ATXN1*	*PPP2R2A*	*ZFHX3*	
				*CNOT6*	*PTEN*	*ZFP36L1*	
	**TGF-β1-stimulated**	Transforming growth factor beta receptor signaling pathway (Biological process)	*TGFBR2 MAP3K1*	*BTG2*	*HAS2*	*SEMA7A*	*ZSWIM6*
				*COL4A5*	*IPMK*	*STK40*	
				*CTDSPL2*	*KBTBD2*	*TMEM64*	
				*DLD*^*c*^	*NGF*^*c*^	*ZFHX3*	
				*GATA6*	*RUNX1*	*ZFP36L1*	
		Wnt signaling pathway (KEGG)	*TCF7L1*	*ACTR10*	*KBTBD2*	*TGFBR2*	
				*CNOT6*	*NPEPPS*	*ZFHX3*	
				*DLD*^*c*^	*POU2F1*	*ZFP36L1*	
				*GATA6*	*PTEN*		
				*HAS2*	*SPRY1*		
		TGF-beta signaling pathway (KEGG)	*TGFBR2*	*BTG2*	*NPEPPS*	*ZFHX3*	
				*DLD*^*c*^	*PLAGL2*	*ZFHX4*	
				*GATA6*	*RUNX1*	*ZFP36L1*	
				*HAS2*	*TCF7L1*	*ZSWIM6*	
				*NGF*^*c*^	*TMEM64*		
**miR-21-3p**	**Unstimulated**	TGF-beta signaling pathway (KEGG)	-	*ATP13A3*	*STK38L*		
				*BTBD7*	*ZBTB39*		
				*KCNA3*	*ZNF217*		
				*NAP1L3*			
				*RHOB*			
		Wnt signaling pathway (KEGG)	-	*ANKRD49*	*SLC19A2*		
				*CDK8*	*SOS2*		
				*GPCPD1*	*TSC22D2*		
				*KCNA3*	*ZBTB39*		
				*RASSF3*	*ZKSCAN3*		
		Signaling by TGF beta (Reactome)	-	*BTBD7*	*RASSF3*	*ZKSCAN3*	
				*ELOVL4*	*SNRK*		
				*KCNA3*	*SOS2*		
				*KIAA1958*	*ZADH2*		
				*MMD*	*ZBTB39*		
	**TGF-β1-stimulated**	TGF-beta signaling pathway (KEGG)	-	*ATP13A3*	*ZBTB39*		
				*BTBD7*	*ZNF217*		
				*KCNA3*			
				*PLSCR4*			
				*STK38L*			
		Signaling by TGF beta (Reactome)	-	*BTBD7*	*MMD*		
				*ELOVL4*	*SOS2*		
				*HHEX*^*c*^	*ZADH2*		
				*KCNA3*	*ZBTB39*		
				*KIAA1958*			

^a^ Annotated genes are genes known to be involved in specific pathways and biological processes.

^b^ Unannotated genes are genes assigned to specific pathways and biological processes by GeneNetwork in which the gene functions were predicted based on co-expression.

^c^ Genes which are more prominently enriched in the IP of TGF-β1-stimulated fibroblasts compared to unstimulated fibroblasts.

Amongst the 55 and 54 predicted miR-21-3p targets present in the miRNA-targetome of unstimulated and TGF-β1-stimulated fibroblasts, respectively, the top 10 most significant biological processes and pathways per collection, are depicted in Table 6. These processes and pathways are related to kinase activity, cancer, cell cycle, metabolism and various signaling pathways. The TGF-β signaling pathway was significantly enriched among the genes with a predicted miR-21-3p binding site both in unstimulated (p≤2x10^-3^) and TGF-β1-stimulated lung fibroblasts (p≤2x10^-3^). Moreover, among the predicted miR-21-3p target genes in the unstimulated lung fibroblasts, the Wnt signaling pathway was also significantly enriched (p = 4x10^-3^). The predicted miR-21-3p targets involved in TGF-β and Wnt signaling pathways were allocated as annotated genes by GeneNetwork (Table 5).

**Table 6:** Most enriched biological processes/pathways within the Ago2-IP enriched and TargetScan predicted miR-21-3p target genes.

	** **	**Pathway/term**	**p-value**
**No TGF-β1**	**Biological process**	Negative regulation of protein kinase activity	9 x 10^−7^
		Peptidyl-threonine modification	3 x 10^−6^
		Negative regulation of kinase activity	4 x 10^−6^
		Negative regulation of transferase activity	5 x 10^−6^
		Retrograde vesicle-mediated transport, Golgi to ER	1 x 10^−5^
		Positive regulation of erythrocyte differentiation	2 x 10^−5^
		Negative regulation of cyclin-dependent protein kinase activity	2 x 10^−5^
		Negative regulation of protein serine/threonine kinase activity	2 x 10^−5^
		Protein ubiquitination	2 x 10^−5^
		Peptidyl-threonine phosphorylation	3 x 10^−5^
	**KEGG**	Small cell lung cancer	4 x 10^−5^
		Pentose and glucuronate interconversions	10 x 10^−4^
		TGF-beta signaling pathway	2 x 10^−3^
		ErbB signaling pathway	2 x 10^−3^
		Drug metabolism—cytochrome P450	3 x 10^−3^
		Fc gamma R-mediated phagocytosis	3 x 10^−3^
		Glycolysis / Gluconeogenesis	4 x 10^−3^
		Wnt signaling pathway	4 x 10^−3^
		Chronic myeloid leukemia	5 x 10^−3^
		Metabolism of xenobiotics by cytochrome P450	5 x 10^−3^
	**Reactome**	Activated TAK1 mediates p38 MAPK activation	9 x 10^−6^
		Signaling by TGF beta	3 x 10^−5^
		Cytosolic tRNA aminoacylation	5 x 10^−5^
		MAP kinase activation in TLR cascade	5 x 10^−5^
		Vitamin B5 (pantothenate) metabolism	6 x 10^−5^
		Toll Like Receptor 5 (TLR5) Cascade	8 x 10^−5^
		Toll Like Receptor 10 (TLR10) Cascade	8 x 10^−5^
		MyD88 cascade initiated on plasma membrane	8 x 10^−5^
		G0 and Early G1	8 x 10^−5^
		Signaling by BMP	1 x 10^−4^
		**Pathway/term**	**p-value**
**TGF-β1**	**Biological process**	Negative regulation of cyclin-dependent protein kinase activity	4 x 10^−7^
		Retrograde vesicle-mediated transport, Golgi to ER	3 x 10^−6^
		Regulation of transcription involved in G1/S phase of mitotic cell cycle	7 x 10^−6^
		Toll-like receptor 1 signaling pathway	8 x 10^−6^
		Toll-like receptor 2 signaling pathway	9 x 10^−6^
		Protein ubiquitination	1 x 10^−5^
		MyD88-dependent toll-like receptor signaling pathway	1 x 10^−5^
		Negative regulation of protein kinase activity	1 x 10^−5^
		Peptidyl-threonine modification	2 x 10^−5^
		Negative regulation of protein serine/threonine kinase activity	2 x 10^−5^
	**KEGG**	Small cell lung cancer	1 x 10^−4^
		Steroid hormone biosynthesis	10 x 10^−4^
		Starch and sucrose metabolism	2 x 10^−3^
		Chronic myeloid leukemia	2 x 10^−3^
		NOD-like receptor signaling pathway	2 x 10^−3^
		TGF-beta signaling pathway	2 x 10^−3^
		Drug metabolism—cytochrome P450	3 x 10^−3^
		Glycolysis / Gluconeogenesis	4 x 10^−3^
		Metabolism of xenobiotics by cytochrome P450	5 x 10^−3^
		Aminoacyl-tRNA biosynthesis	6 x 10^−3^
	**Reactome**	G0 and Early G1	8 x 10^−6^
		Signaling by TGF beta	9 x 10^−6^
		Interleukin-1 signaling	2 x 10^−5^
		Toll Like Receptor 2 (TLR2) Cascade	2 x 10^−5^
		MyD88:Mal cascade initiated on plasma membrane	2 x 10^−5^
		Toll Like Receptor TLR6:TLR2 Cascade	2 x 10^−5^
		Toll Like Receptor TLR1:TLR2 Cascade	2 x 10^−5^
		Toll Like Receptor 5 (TLR5) Cascade	4 x 10^−5^
		Toll Like Receptor 10 (TLR10) Cascade	4 x 10^−5^
		MyD88 cascade initiated on plasma membrane	4 x 10^−5^

### Identification of target genes modulated by TGF-β1-induced miR-455-3p and miR-21-3p

Next, we investigated which target genes are affected by stimulation with TGF-β1. These target genes are defined as being more prominently enriched in the IP of TGF-β1-stimulated fibroblasts compared to unstimulated fibroblasts. Five miR-455-3p predicted target genes *HN1*, *NGF*, *STRADB*, *DLD* and *ANO3* showed an increased enrichment in the IP upon TGF-β1 stimulation. Of these genes, *NGF* and *DLD* were predicted by GeneNetwork to be involved in the TGF-β1-related biological processes and signaling pathway (marked bold in Table 5). Furthermore, *DLD* is predicted to play a role in the Wnt signaling pathway.

Of the predicted miR-21-3p target genes, increased Ago2-IP enrichment upon TGF-β1 stimulation was observed for *HHEX*, *CHORDC1* and *ZBTB49*. *HHEX* was one of the genes predicted to be involved in the TGF-β signaling pathway (marked bold in Table 5).

## Discussion

In the present study, we investigated the effect of TGF-β1 on miRNA expression in primary lung fibroblasts and used the miRNA-targetomes of these cells to identify transcripts that are likely to be affected by the TGF-β1-dependent modulation of miRNA expression levels. Twenty-nine miRNAs were differentially expressed after TGF-β1 stimulation. TGF-β1-induced miR-455-3p and miR-21-3p expression was validated with qRT-PCR and also replicated in an independent set of control and COPD fibroblasts. In the replication set, lower levels of miR-455-3p were observed in COPD as compared to control fibroblasts. The TGF-β signaling pathway was significantly enriched amongst the Ago2-IP enriched predicted targets of miR-455-3p and miR-21-3p and we have identified several target genes, which were more Ago2-IP-enriched upon TGF-β1 stimulation.

We showed that TGF-β1 regulates expression of multiple miRNAs in primary lung fibroblasts of control subjects and COPD patients with consistent and significant induction of miR-455-3p and miR-21-3p. In a previous study of Milosevic et al., 84 miRNAs were shown to be differentially expressed after TGF-β1 stimulation in commercially available human lung fibroblasts [27]. Seven out of the 29 differentially expressed miRNAs in our initial miRNA array screening were overlapping with the 84 miRNAs found by Milosevic et al. In accordance with their results, we showed upregulation of miR-21-3p, miR-23a-5p, miR-503-5p, miR-424-3p, and miR-214-5p upon TGF-β1 stimulation. However, they found downregulation of miR-455-3p and miR-23a-3p [27], whereas we found upregulation of these two miRNAs. These inconsistencies might be due to differences in using a single versus multiple donors, culture methods and TGF-β1 stimulation time (2 h versus 24 h). Notably, upregulation of miR-455-3p and miR-23a-3p after TGF-β stimulation has been observed in other cell types [28–31]. Other studies observed increased expression levels of miR-27a-3p and miR-199a-5p upon TGF-β stimulation in MRC-5 lung fibroblasts in accordance with our initial miRNA array results [8, 32].

Using Ago2-RIP-Chip, we defined the miRNA-targetomes of both unstimulated and TGF-β1-stimulated primary lung fibroblasts. By assessing which of the miR-455-3p and miR-21-3p predicted targets are present in these targetomes we gained insight in the potential role of these TGF-β1-induced miRNAs in lung fibroblasts. To our knowledge this is the first time that the miRNA-targetomes have been identified in primary lung fibroblasts. The lists of experimentally proven targets of miRNAs in primary lung fibroblasts with and without TGF-β1 stimulation represent a valuable source for future studies. The predicted miR-455-3p and miR-21-3p targets present in the miRNA-targetomes of unstimulated and TGF-β1-stimulated lung fibroblasts were significantly enriched for genes related to the TGF-β processes and signaling pathway. These findings suggest that there is significant cross-talk between miRNAs and the TGF-β pathway. Moreover, the enrichment of the TGF-β signaling pathway suggests a role for these miRNAs in regulating ECM homeostasis by lung fibroblasts [6].

Previous data suggested that the TGF-β1-induced miR-455-3p expression is regulated through a SMAD2/3 binding element and that miR-455-3p directly targets *SMAD2*, *ACVR2B* and *CHRDL1* [31]. This suggests a feedback loop resulting in decreased SMAD2/3 signaling. From these reported targets of miR-455-3p, *ACVR2B* was found in the miRNA-targetome of unstimulated primary fibroblasts. *SMAD2* and *CHRDL1* were expressed in primary lung fibroblasts but were not enriched in the IP fractions. Of the IP-enriched and predicted miR-455-3p target genes, the levels of enrichment of *DLD* and *NGF* transcripts in the Ago2-IP fraction were increased upon TGF-β1 stimulation. Both genes were predicted by GeneNetwork to play a role in the TGF-β-related processes and signaling pathway. DLD is a sub-protein of the pyruvate dehydrogenase protein complex, which is involved in the cellular metabolism [33]. Interestingly, pyruvate dehydrogenase activity was found to be decreased in the muscles of COPD patients [33]. NGF is a neurotrophin which can be produced and released by lung fibroblasts during inflammation. A previous study showed that NGF accelerates the migration of human fetal lung fibroblasts to fibronectin indicating that NGF may stimulate repair [34]. Furthermore, NGF itself and TGF-β enhance the NGF release by MRC-5 cells and NGF, similarly to TGF-β, induces α-SMA expression in these cells [35]. The same study showed that expression of NGF and its receptors, trkA and p75, was increased in fibrotic lung tissue compared to normal tissue [35]. Taken together, NGF may have pro-fibrotic effects in the lung and TGF-β1-induced miR-455-3p may serve to control NGF production inhibiting its pro-fibrotic effects on lung fibroblasts.

A second gene set enriched within the Ago2-IP-enriched miR-455-3p targets is the Wnt signaling pathway, with *TCF7L1* being a predicted target and present in the miRNA-targetomes of both unstimulated and TGF-β1-stimulated lung fibroblasts. TCF7L1 mediates the transcription of the canonical Wnt signaling target genes [36]. *DLD* was a second gene predicted to be involved in the Wnt signaling pathway. The Wnt pathway regulates the differentiation of lung fibroblasts into myofibroblasts after activation via TGF-β and this enhances the ECM production [37]. Therefore, activation of the Wnt signaling pathway has been suggested to be an important factor in tissue repair. Deregulation of this pathway via miRNAs may thus contribute to the disturbed ECM homeostasis in lung diseases.

The lower expression level of miR-455-3p in COPD patients compared to control subjects indicates its potential role in aberrant tissue repair and remodeling of the lungs via effects on the target genes involved in the Wnt and TGF-β signaling pathways.

As mentioned above, the TGF-β signaling pathway was also enriched amongst the IP-enriched and predicted miR-21-3p target genes. *HHEX* is one of the genes whose Ago2-IP enrichment increases upon TGF-β1 stimulation and predicted to be involved in the TGF-β signaling pathway. The expression of this homeobox containing gene was found to be regulated by the BMP signaling pathway and is implied to be involved in lung organogenesis [38].

Other IP-enriched and predicted miR-21-3p target genes that were more enriched after TGF-β1 stimulation are *CHORDC1* and *ZBTB49*. *CHORDC1* has been shown to inhibit kinase activity of ROCK2 in mouse embryonic fibroblasts [39]. Rho kinases have been implicated in asthma and COPD and have been suggested as a promising therapeutic target for these lung diseases [40]. *ZBTB49* is a zinc finger that can inhibit cell proliferation of HEK293 and HCT116 cells and thus may affect cell proliferation in lung fibroblasts as well [41].

## Conclusions

We demonstrated that TGF-β1 affects the expression of several miRNAs in primary parenchymal lung fibroblasts, including miR-455-3p and miR-21-3p. Furthermore, we showed that the targets of miR-455-3p and miR-21-3p play a role in the TGF-β and Wnt signaling pathways. In addition, our study showed that TGF-β1-dependent miRNA modulation affects several genes that may influence the function of lung fibroblasts via several processes including tissue repair, cellular metabolism, cell migration and cell proliferation. Interestingly, we found a difference in miR-455-3p expression between COPD patients and control subjects, which suggests a role for this miRNA in COPD pathogenesis. These findings add to our understanding of the role of miRNAs in the functionality of lung fibroblasts in tissue repair and remodeling and support a role for aberrant miRNA regulation in lung diseases such as COPD in which high TGF-β levels have been reported.

## Supporting information

S1 Tables Top 1,500 Ago2-IP-enriched probes of each IP experiment and the identified genes in the miRNA-targetomes. The top 1,500 most IP-enriched probes of each of the four IP experiments are ranked based on the IP/T ratios. The miRNA-targetomes of the unstimulated and TGF-β1-stimulated lung fibroblasts were defined as the overlap of the top 1,500 most IP-enriched probes in the fibroblasts of the two control subjects (see Fig 5).
(XLSX)
